# Prevalence and factors associated with non-use of health services in the Peruvian population with COVID-19 symptomatology: a secondary analysis of the 2020 National Household Survey

**DOI:** 10.4178/epih.e2021084

**Published:** 2021-10-18

**Authors:** Akram Hernández-Vásquez, Fabriccio J. Visconti-Lopez, Diego Azañedo

**Affiliations:** 1 Centro de Excelencia en Investigaciones Económicas y Sociales en Salud, Vicerrectorado de Investigación, Universidad San Ignacio de Loyola, Lima, Peru; 2 Universidad Peruana de Ciencias Aplicadas, Lima, Peru; 3 Universidad Científica del Sur, Lima, Peru

**Keywords:** COVID-19, Health services accessibility, Health systems, Peru

## Abstract

**OBJECTIVES:**

The objective of this study was to estimate the prevalence of non-use of health services (NUHS) and its associated factors in Peruvians with symptoms of coronavirus disease 2019 (COVID-19).

**METHODS:**

A secondary analysis of the 2020 National Household Survey (ENAHO) was carried out. Participants over 18 years of age with any COVID-19 symptom (fever, cough, sensation of shortness of breath) in the last 4 weeks who did not visit health services were defined as exhibiting NUHS. Adjusted prevalence ratios (aPRs) were estimated to determine the factors associated with NUHS.

**RESULTS:**

Data from 1,856 participants were analyzed; the prevalence of NUHS was 52.2% (95% confidence interval [CI], 48.0 to 56.5). Living in urban areas of the jungle (aPR, 1.61; 95% CI, 1.32 to 1.98; p<0.001) and rural areas of the jungle (aPR, 1.48; 95% CI, 1.15 to 1.90; p=0.002) was associated with a higher probability of NUHS than living in urban coastal areas. The factors associated with a lower probability of NUHS were being 50-59 years old (aPR, 0.72; 95% CI, 0.58 to 0.90) and 60 years and over (aPR, 0.74; 95% CI, 0.59 to 0.95), having a secondary educational level (aPR, 0.67; 95% CI, 0.48 to 0.93) or superior educational level (aPR, 0.67; 95% CI, 0.48 to 0.96), and having health insurance (aPR, 0.79; 95% CI, 0.68 to 0.92).

**CONCLUSIONS:**

More than half of the participants with COVID-19 symptoms did not use health services, and NUHS was associated with the geographic and socio-demographic characteristics of the population. The formulation of health strategies and programs is required to increase the use of health services by people with COVID-19 symptoms.

## INTRODUCTION

On March 11, 2020, the World Health Organization (WHO) declared coronavirus disease 2019 (COVID-19) a pandemic, due to its rapid global spread and mortality [1]. As of the end of June 2021, there have been more than 183.4 million confirmed cases of COVID-19 and more than 3.9 million deaths have been reported from this disease in 207 countries around the world [2,3]. Peru is the country with the 18th most cases worldwide, and it is also the country with the highest case-fatality rate (9.4%) [2,3]. Additionally, the impact of the pandemic has been particularly intense in Peru, since it is a developing country, with a medium response capacity to pandemics, limited access to basic services, high rates of poverty and job insecurity, and public spending on health accounting for 4.2% of the gross domestic product (GDP) in 2020, a value below WHO recommendations (6.0% of GDP) [4]; these factors mainly affect disadvantaged populations in Peru [5,6].

The government of Peru took measures to try to control the collapse of the health system in the country [7], such as implementing a state of national emergency, mandatory quarantine, and border closures; however, these measures did not have the expected success. Thus, at present, Peru registers a figure of 5,551 deaths from COVID-19 per million inhabitants, which is the highest rate in the world for this indicator [8]. This could be due to the existing limitations in the Peruvian health system, which had only 525 intensive care unit (ICU) beds at the beginning of the pandemic and 13.6 doctors per 10,000 inhabitants, 9.4 less than the proportion recommended by the WHO [9,10]. Currently, 2,678 ICU beds with mechanical ventilators have been installed, but this is still a relatively small number for 33 million inhabitants [11]. In addition, compliance with government measures to combat the spread of the pandemic has not been high [12].

During the COVID-19 pandemic, the use of health services at different levels of care has been very limited worldwide [13]. In 2020, the Ministry of Health (MINSA) of Peru recommended that people presenting symptoms such as fever or respiratory distress should go to the health center closest to their home for a professional evaluation, and if necessary, undergo a COVID-19 rule-out test [14]. However, several characteristics that shape the population’s access to healthcare may have influenced the search for professional care among the Peruvian population with symptoms of COVID-19. Some studies and access models have reported that factors such as education, ethnicity, sex, geographical conditions, having health insurance, self-medication, number of nearby facilities, the perception of not considering the symptoms of severity, and the fear of being diagnosed with a serious disease were associated with non-use of health services (NUHS) [15-19]. However, although previous studies have evaluated these associations, given the imminent collapse of the health system and the resultant needs, no study has evaluated this association within the context of the COVID-19 pandemic in Peru.

Therefore, the objective of this study was to identify the determinants of the NUHS in Peruvians with COVID-19 symptoms, using information from the 2020 National Household Survey. We believe that our results could be useful for the development of health strategies and programs to improve access to health services in the Peruvian population with symptoms of COVID-19.

## MATERIALS AND METHODS

### Design and study population

A cross-sectional analytical study was carried out using secondary data from the 2020 National Household Survey on Living Conditions and Poverty (ENAHO), prepared by the National Institute of Statistics and Informatics (INEI). The ENAHO is a representative survey at the level of the Peruvian population that is carried out annually and collects information from members of Peruvian households on education, health, employment and income, household expenses and opinions on governance, democracy, discrimination, perceptions of insecurity, and corruption, among others (https://webinei.inei.gob.pe/anda_inei/index.php/catalog/714).

The ENAHO 2020 was a cross-sectional survey that used probabilistic, area-based, stratified, multi-stage, and independent sampling with representativeness at the national, departmental, natural region, and urban/rural levels. Data collection of the ENAHO 2020 during the period of social isolation was carried out through telephone interviews (from March 16 to September 30, 2020) and later through face-to-face interviews (from October to December 2020) supplemented with telephone call interviews that followed a standard procedure with training of survey personnel, pilot tests, supervision of surveyors, and data quality control. The ENAHO 2020 sample size was 37,103 households, of which 23,895 households were in urban areas and 13,208 were in rural areas. The National Institute of Statistics and Informatics has published technical documents related to the design, instruments, procedures, and manuals of ENAHO 2020, which can be accessed through the “Documentation” tab at the following link http://iinei.inei.gob.pe/microdatos/.

**Figure 1.**
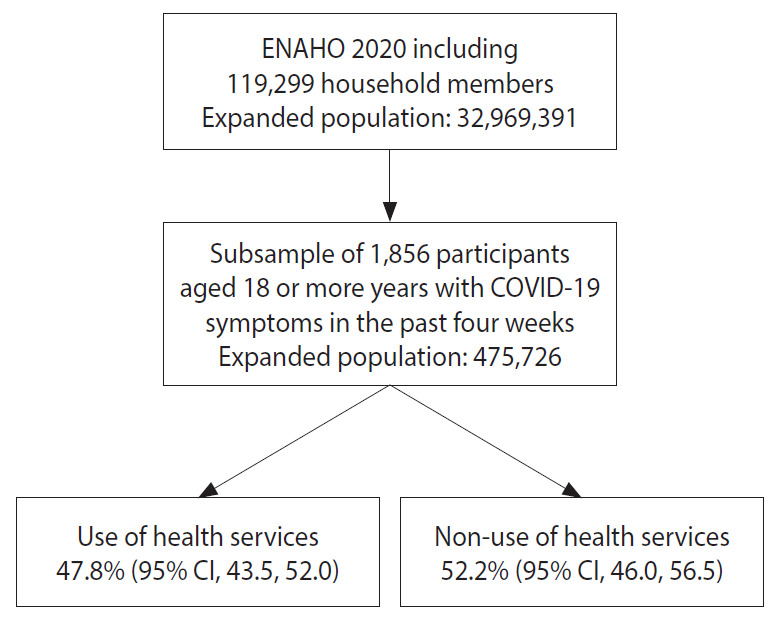
Flow chart of the participants included in the study. ENAHO, National Household Survey on Living Conditions and Poverty; COVID-19, coronavirus disease 2019; CI, confidence interval.

For the present study, a subsample of adult participants aged 18 years or older who reported having experienced a COVID-19 symptom (fever, cough, sensation of shortness of breath) in the last 4 weeks was considered (n=1,856). Details of the selection of the subsample included are presented in Figure 1.

### Variables and measurements

The dependent variable was NUHS in Peruvian adults with symptoms of COVID-19 in the last 4 weeks prior to the survey. This variable was created based on responses to the question, “Where did you go to consult for this disease, symptom, discomfort, and/or accident?” The responses were classified as 1 “did not use health services” and 0 “used health services.” The category “did not use health services” included not having attended a health establishment operated by the MINSA, social security (EsSalud), the Armed Forces and National Police of Peru, or private clinics and clinics, and the category “used health services” included instances where the individual did attend a health establishment after experiencing a symptom of COVID-19 (fever, cough, sensation of shortness of breath) in the last 4 weeks. The variable was created using references to previous studies carried out in Peru [19,20].

The following independent variables were included: sex (male or female), age group (18-29, 30-39, 40-49, 50-59, or 60 or older), educational level (no education, primary, secondary, or superior education), marital status (with or without a partner), ethnic self-identification (non-native or native), suffering from a chronic disease (yes or no), presence of any physical or psychological or cognitive limitation (yes or no), health insurance coverage (yes or no), the presence of an unsatisfied basic need (yes or no) and geographic domain of residence (urban coastal areas, rural coastal areas, urban highland areas, rural highland areas, urban areas of the jungles, rural areas of the jungle, or Metropolitan Lima). These independent variables were selected due to their positive association with access to health services as found in previous studies and taking into account the Andersen model [16-20].

### Statistical analysis

For data analysis, the statistical program Stata version 14.2 (StataCorp., College Station, TX, USA) was used. All statistical tests were considered significant if the p-value was less than 0.05. The complex design of the ENAHO 2020 sampling was adjusted using the command “svyset [pw=factor07], psu (conglome) strata (estrato)”

Descriptive and inferential analyses were carried out for the subsample of interest using the “subpop” option of the Stata program. In the descriptive analysis, the socio-demographic characteristics of the study population were described using weighted frequencies and proportions. NUHS was analyzed according to the socio-demographic characteristics of the study population. An inferential analysis of the factors associated with the NUHS in Peruvian adults with COVID-19 symptoms was performed using a generalized linear model of the Poisson family and logarithmic link to obtain crude prevalence ratios (PRs) and adjusted PRs (aPR) together with their 95% confidence intervals (CIs). Variables with a p-value < 0.20 in the crude analysis were included in the adjusted analysis. Multicollinearity of the independent variables was evaluated using the variance inflation factor (VIF) using the command “collin”.

### Ethics statement

The study did not require the approval of an ethics committee as it was an analysis of secondary data available in the public domain from which it is not possible to identify the participants. All ENAHO 2020 data are completely anonymized, de-identified, and/or aggregated. The databases used are freely accessible on the INEI web portal (http://iinei.inei.gob.pe/microdatos/).

## RESULTS

Of a total of 1,856 participants with COVID-19 symptoms in the last 4 weeks, 52.2% (95% CI, 48.0 to 56.5) did not go to a health service. Most of the participants were females (51.4%), in the age group of 50-59 years (21.8%), had reached a secondary educational level (42.7%), and had a partner (61.8%); likewise, the majority self-identified as non-native (78.1%), 55.3% of the participants had a chronic disease, and 94.0% had a physical, psychological or cognitive limitation. Furthermore, 73.1% of those surveyed had health insurance, and 15.0% had an unsatisfied basic need. The highest proportion of participants lived in Metropolitan Lima (41.3%), followed by urban coastal areas (21.6%), urban areas of the jungle (15.7%) and urban highland areas (12.2%) (Table 1).

There were significant differences in the frequency of use and NUHS in patients with symptoms of COVID-19 according to the following categorical variables: having health insurance (p=0.008), the presence of an unsatisfied basic need (p=0.016), and geographic domain (p<0.001). More specifically, over 6 out of 10 (61.3%; 95% CI, 52.9 to 69.0) participants who did not have health insurance did not go to a health service. Similarly, 62.2% (95% CI, 54.1 to 69.7) of those who had an unsatisfied basic need also did not go to a health service despite having symptoms of COVID-19. Similarly, 69.9% (95% CI, 62.7 to 76.3) of participants from urban areas of the jungle and 68.4% (95% CI, 57.6 to 77.5) from rural areas of the jungle did not go to a health service despite having symptoms of COVID-19 (Table 1).

In the crude model, the factors associated with a greater probability of not using health services in patients with COVID-19 symptoms were having an unsatisfied basic need (PR, 1.23; 95% CI, 1.05 to 1.45; p=0.011), compared to not having any unsatisfied basic needs; and residing in urban areas of the jungle (PR, 1.65; 95% CI, 1.36 to 2.01; p<0.001) or rural areas of the jungle (PR, 1.62; 95% CI, 1.29 to 2.03; p<0.001), compared to those who resided in urban coastal areas. Additionally, the factors associated with a lower probability of not using health services were being 50-59 years of age (PR, 0.75; 95% CI, 0.60 to 0.92; p=0.007), compared to the age group of 18-29 years old; and having health insurance (PR, 0.80; 95% CI, 0.68 to 0.93; p=0.004), compared to not having health insurance (Table 2).

In the adjusted model, the factors associated with a greater probability of not using health services in patients with COVID-19 symptoms were residing in urban areas of the jungle (aPR, 1.61; 95% CI, 1.32 to 1.98; p<0.001) and rural areas of the jungle (aPR, 1.48; 95% CI, 1.15 to 1.90; p=0.002), compared to those residing in urban coastal areas. Likewise, the factors associated with a lower probability of not using health services were being 50-59 years old (aPR, 0.72; 95% CI, 0.58 to 0.90; p=0.004), and 60 years and over (aPR, 0.74; 95% CI, 0.59 to 0.95; p=0.016), compared with those who were 18-29 years old; having a secondary educational level (aPR, 0.67; 95% CI, 0.48 to 0.93; p=0.018) or superior educational level (aPR, 0.67; 95% CI, 0.48 to 0.96; p=0.027), compared to those without formal education; and having health insurance (aPR, 0.79; 95% CI, 0.68 to 0.92; p=0.003), compared to those without health insurance.

## DISCUSSION

The present study identified that 5 out of 10 Peruvians did not go to a health center despite presenting symptoms of COVID-19 in the 4 weeks prior to their participation in the survey. This is a low percentage considering that in 2020 the MINSA of Peru recommended that people presenting signs or symptoms of COVID-19 (including the sensation of shortness of breath and fever greater than 38°C) should go to the health center closest to their home for a medical evaluation and, if necessary, undergo a COVID-19 rule-out test. This is relevant, since 2 of the 3 symptoms mentioned in the survey were the presence of fever and the sensation of shortness of breath, which have been reported as prognostic factors of greater severity of COVID-19 [21]. This situation is not alien to other latitudes. In Korea, a study reported that 73.2% of study participants avoided using health services during the pandemic [22]. On the other hand, in China, hospital visits for any cause fell by 51% after the COVID-19 pandemic [23]. These figures indicate that the NUHS is a problem that is probably affecting most low-income and middle-income countries worldwide due to the pandemic.

Respondents from rural and urban jungle areas with COVID-19 symptoms had a higher probability of not using health services than residents of the urban coast. Although no comparable studies have been conducted in Peru in the context of the COVID-19 pandemic, a similar situation was previously reported in 2015, in which residents of the Peruvian jungle were more likely not to use formal health services in the presence of any symptoms, discomfort, illness, relapse of chronic illness, or accident during the last month prior to being surveyed [19]. This problem is not exclusive to health services in general, since it has also been reported that living in the jungle was associated with lower probabilities of using dental services, both in children and older adults, compared to residents of Metropolitan Lima, which is an urban coastal area [24,25]. This may be due to the fact that most of the regions that comprise the Peruvian jungle have the smallest number of health facilities at the national level [26,27], which may limit opportunities to access for the population. Furthermore, geographic accessibility and ease of transportation to a health facility may be limited in this geographic region [28]. In 2013, it was reported that the average time that residents of rural areas took to travel on foot to the nearest health center was 34 minutes, compared to 24 minutes on the coast, while the average time for transit using motorized transportation was 32 minutes in the jungle, compared to 16 minutes on the coast [29]. Taking this into account, it is important to facilitate and guarantee timely care to patients with symptoms of COVID-19 in the jungle of Peru, which is a region that has been strongly affected by the pandemic.

The Peruvian elderly population aged 50-59 years and 60 years and over with symptoms of COVID-19 showed a lower likelihood of not using health services than the population aged 18-29 years. This could be attributed to the fact that older adults are considered a population at greater risk for severe COVID-19, mainly due to the higher prevalence of comorbidities, such as diabetes, cardiovascular diseases, and obesity, which may explain why this group was more likely to seek care. Additionally, constant information in the mass media may have generated changes in perceptions of COVID-19, modifications in self-care for health, and care-seeking behavior in the presence of COVID-19 symptoms in this particularly vulnerable group [30]. Nevertheless, it is important to increase care-seeking behavior in younger populations, because despite having a lower risk of severe COVID-19, they represent a large proportion of the working and socially active population, and are an important part of the severe acute respiratory syndrome coronavirus 2 (SARS-CoV-2) transmission chain. Furthermore, a study in China reported that patients over 60 years of age took an average of 2.5 days longer than patients under 30 years to seek medical attention from the onset of symptoms suggestive of COVID-19. Therefore, it is necessary to seek to improve access to timely care in this group of particular vulnerability [31].

Peruvians with symptoms of COVID-19 with a secondary and superior educational level were less likely not to use health services than those without a formal education. In this regard, it has been reported that having a higher educational level is significantly associated with greater knowledge, positive attitudes, and good practices regarding the prevention of the spread of COVID-19 [32]. The above could explain what happened in the Peruvian population. Thus, those who presented symptoms of COVID-19 may have used services with the intention of ruling out the disease and taking the necessary isolation measures if they had COVID-19 to prevent transmission of the disease to their relatives or friends.

Furthermore, Peruvians who had health insurance were less likely not to use a health service in the presence of COVID-19 symptoms than those without health insurance. A similar situation has been reported in a study of females participating in a national representative survey in the United States, in which 45% of those with private insurance, and 41% of those insured with Medicaid underwent COVID-19 testing, compared with 28% of surveyed females who did not have health insurance [33]. This could occur because having health insurance can be associated with positive perceptions regarding access and availability of diagnostic tests for COVID-19 [34]. Instead, those with confirmed symptoms and COVID-19, but without health insurance, may think that going to a public or private health service for an evaluation and medical care would lead to them either not being seen or having to pay large amounts of money for care.

This study has limitations inherent to its design. One limitation is that its cross-sectional design does not allow causality to be determined. Similarly, there is the possibility of memory bias, because the measurement of the outcome of interest was based on a question about having presented symptoms of COVID-19 in the 4 weeks prior to the survey. In addition, since this was a secondary data analysis, the factors evaluated were limited to the variables available in the survey, although there may have been other influencing factors, such as perceptions regarding COVID-19 or knowledge regarding how to seek out medical care due to disease, which were not analyzed due to their unavailability in the dataset. However, this study preliminarily identified the factors associated with the NUHS in the Peruvian population presenting COVID-19 symptoms in the last 4 weeks. These findings should be studied in greater depth in future prospective studies on the subject.

In conclusion, just over half of the Peruvian population with symptoms of COVID-19 participating in the ENAHO 2020 did not use health services, despite the recommendations of the MINSA. Likewise, geographic and socio-demographic factors associated with the NUHS were identified. NUHS in the Peruvian population could generate a delay in the detection of COVID-19 cases that could have an unfavorable prognosis, generating a delay in timely care for these patients. Similarly, it is likely that undiagnosed cases do not take adequate isolation measures, thereby transmitting COVID-19 to their family and people in their social and work environments, consequently leading to saturation of health services. Therefore, it is necessary to formulate and implement public health measures that address this problem and take into account the associated factors in order to increase the use of health services.

## Figures and Tables

**Table 1.** Characteristics of the participants included in the study^1^

Characteristics		n (%)	Utilization of health services in persons with COVID-19 symptomatology		
			Yes	No	p-value
Overall		1,856 (100)	47.8 (43.5, 52.0)	52.2 (48.0, 56.5)	
Sex					
	Male	888 (48.6)	49.3 (43.8, 54.9)	50.7 (45.1, 56.2)	0.393
	Female	968 (51.4)	46.3 (40.9, 51.7)	53.7 (48.3, 59.1)	
Age (yr)					
	18-29	370 (19.9)	40.9 (32.7, 49.7)	59.1 (50.3, 67.3)	0.049
	30-39	418 (21.6)	45.1 (37.9, 52.5)	54.9 (47.5, 62.1)	
	40-49	415 (21.1)	45.2 (37.6, 53.0)	54.8 (47.0, 62.4)	
	50-59	359 (21.8)	55.9 (47.6, 63.8)	44.1 (36.2, 52.4)	
	≥60	294 (15.7)	52.4 (43.8, 60.8)	47.6 (39.2, 56.2)	
Educational level					
	No education	48 (2.4)	35.5 (20.5, 53.9)	64.5 (46.1, 79.5)	0.135
	Primary	406 (19.7)	40.8 (33.2, 48.9)	59.2 (51.1, 66.8)	
	Secondary	728 (42.7)	49.8 (43.4, 56.3)	50.2 (43.7, 56.6)	
	Superior	674 (35.2)	50.0 (43.8, 56.2)	50.0 (43.8, 56.2)	
Partner					
	Without partner	698 (38.2)	46.1 (39.7, 52.7)	53.9 (47.3, 60.3)	0.519
	With Partner	1,158 (61.8)	48.8 (43.6, 53.9)	51.2 (46.1, 56.4)	
Ethnicity					
	Not native	1,435 (78.1)	46.6 (41.9, 51.4)	53.4 (48.6, 58.1)	0.243
	Native	421 (21.9)	51.8 (44.1, 59.4)	48.2 (40.6, 55.9)	
Presence of any chronic disease					
	No	870 (44.7)	44.9 (39.1, 50.8)	55.1 (49.2, 60.9)	0.164
	Yes	986 (55.3)	50.1 (44.7, 55.4)	49.9 (44.6, 55.3)	
Presence of any physical or psychological or cognitive limitation					
	No	1,757 (94.0)	48.0 (43.7, 52.3)	52.0 (47.7, 56.3)	0.575
	Yes	99 (6.0)	43.8 (30.0, 58.6)	56.2 (41.4, 70.0)	
Health insurance					
	No	415 (26.9)	38.7 (31.0, 47.1)	61.3 (52.9, 69.0)	0.008
	Yes	1,441 (73.1)	51.1 (46.5, 55.7)	48.9 (44.3, 53.5)	
Some unsatisfied basic need					
	No	1,507 (85.0)	49.5 (44.7, 54.3)	50.5 (45.7, 55.3)	0.016
	Yes	349 (15.0)	37.8 (30.3, 45.9)	62.2 (54.1, 69.7)	
Geographic domains					
	Urban coast	513 (21.6)	57.7 (50.3, 64.7)	42.3 (35.3, 49.7)	<0.001
	Rural coast	35 (0.7)	57.5 (37.9, 75.0)	42.5 (25.0, 62.1)	
	Urban highlands	202 (12.2)	57.6 (45.6, 68.8)	42.4 (31.2, 54.4)	
	Rural highlands	103 (3.4)	45.9 (35.5, 56.6)	54.1 (43.4, 64.5)	
	Urban jungle	511 (15.7)	30.1 (23.7, 37.3)	69.9 (62.7, 76.3)	
	Rural jungle	219 (5.0)	31.6 (22.5, 42.4)	68.4 (57.6, 77.5)	
	Metropolitan Lima	273 (41.3)	48.4 (40.3, 56.5)	51.6 (43.5, 59.7)	

Values are presented as % (95% confidence interval). COVID-19, coronavirus disease 2019; ENAHO, National Household Survey on Living Conditions and Poverty.

1 Estimates include the weights and ENAHO 2020 sample specifications.

**Table 2.** Crude and adjusted prevalence ratios of non-utilization of health services in persons with COVID-19 symptomatology^1^

Variables		Crude	p-value	Adjusted	p-value
Sex					
	Male	1.00 (reference)		Not included	
	Female	1.06 (0.93, 1.21)	0.394		
Age (yr)					
	18-29	1.00 (reference)		1.00 (reference)	
	30-39	0.93 (0.76, 1.13)	0.465	0.94 (0.77, 1.14)	0.506
	40-49	0.93 (0.77, 1.12)	0.436	0.92 (0.76, 1.11)	0.401
	50-59	0.75 (0.60, 0.92)	0.007	0.72 (0.58, 0.90)	0.004
	≥60	0.81 (0.65, 1.01)	0.056	0.74 (0.59, 0.95)	0.016
Educational level					
	No education	1.00 (reference)		1.00 (reference)	
	Primary	0.92 (0.69, 1.23)	0.559	0.84 (0.61, 1.16)	0.293
	Secondary	0.78 (0.58, 1.04)	0.091	0.67 (0.48, 0.93)	0.018
	Superior	0.78 (0.58, 1.04)	0.088	0.67 (0.48, 0.96)	0.027
Partner					
	Without partner	1.00 (reference)		Not included	
	With Partner	0.95 (0.82, 1.11)	0.516		
Ethnicity					
	Not native	1.00 (reference)		Not included	
	Native	0.90 (0.76, 1.08)	0.256		
Presence of chronic disease					
	No	1.00 (reference)		1.00 (reference)	
	Yes	0.91 (0.79, 1.04)	0.163	0.97 (0.85, 1.12)	0.708
Presence of any physical or psychological or cognitive limitation					
	No	1.00 (reference)		Not included	
	Yes	1.08 (0.83, 1.41)	0.559		
Health insurance					
	No	1.00 (reference)		1.00 (reference)	
	Yes	0.80 (0.68, 0.93)	0.004	0.79 (0.68, 0.92)	0.003
Some unsatisfied basic need					
	No	1.00 (reference)		1.00 (reference)	
	Yes	1.23 (1.05, 1.45)	0.011	1.03 (0.87, 1.23)	0.700
Geographic domains					
	Urban coast	1.00 (reference)		1.00 (reference)	
	Rural coast	1.00 (0.62, 1.64)	0.985	0.98 (0.59, 1.60)	0.923
	Urban highlands	1.00 (0.72, 1.39)	0.993	0.95 (0.70, 1.28)	0.740
	Rural highlands	1.28 (0.98, 1.66)	0.066	1.23 (0.95, 1.59)	0.119
	Urban jungle	1.65 (1.36, 2.01)	<0.001	1.61 (1.32, 1.98)	<0.001
	Rural jungle	1.62 (1.29, 2.03)	<0.001	1.48 (1.15, 1.90)	0.002
	Metropolitan Lima	1.22 (0.97, 1.54)	0.095	1.25 (0.99, 1.59)	0.062

Values are presented as prevalence ratio (95% confidence interval). COVID-19, coronavirus disease 2019; ENAHO, National Household Survey on Living Conditions and Poverty.

1 Estimates include the weights and ENAHO 2020 sample specifications.
